# Umbilical cord mesenchymal stem cell-derived exosomes promote wound healing and skin regeneration via the regulation of inflammation and angiogenesis

**DOI:** 10.3389/fbioe.2025.1641709

**Published:** 2025-11-03

**Authors:** Yulin Yang, Yinfu Huang, Jian Yang, Zhiwei Hu, Shiduo Wu, Qian Yuan, Shuo Meng, Duanduan Li, Meiling Jiang, Yan Liao, Cheguo Cai

**Affiliations:** ^1^ Shenzhen Beike Biotechnology Co., Ltd., Shenzhen, China; ^2^ Shenzhen Beike Biotechnology Research Institute, Shenzhen, China; ^3^ Key Laboratory of Systems Health Science of Zhejiang Province, School of Life Science, Hangzhou Institute for Advanced Study, University of Chinese Academy of Sciences, Hangzhou, China

**Keywords:** exosomes, human umbilical cord mesenchymal stem cells, wound healing, vascularization, regeneration, miRNA

## Abstract

**Introduction:**

Wound healing and skin regeneration have become global health challenges, causing substantial harm to the physical and mental health. Many studies have shown that human umbilical cord mesenchymal stem cell-derived exosomes (hUCMSC-Exos) can promote tissue repair and regeneration. However, the efficacy and underlying mechanisms of hUCMSC-Exos in treatment remain to be elucidated.

**Methods:**

hUCMSC-Exos were isolated by ultracentrifugation and characterized by analyses of nanoparticle tracking analysis (NTA), western blotting (WB), and transmission electron microscopy (TEM). The efficacy of hUCMSC-Exos on the proliferation, migration, and angiogenesis potential of fibroblasts and endothelial cells were examined *in vitro*. The effects of the hUCMSC-Exos on wound healing were assessed by wound closure rate, histological and immunohistochemical analyses. miRNAs and their target genes that may play a role in skin repair and regeneration were identified and predicted through bioinformatics analysis.

**Results:**

*In vitro* analysis indicated that hUCMSC-Exos are easily internalized by human umbilical vein endothelial cells (HUVECs) and human skin fibroblasts (HSFs), significantly promoting the proliferation and migration of HSFs, as well as the proliferation and tube formation of HUVECs. Analysis of skin wound models indicated that hUCMSC-Exos significantly accelerate wound healing by reducing inflammation, stimulating angiogenesis, and promoting the formation of extracellular matrix. Mechanistically, bioinformatics analysis suggests that Unc-51-like autophagy activating kinase 2 (ULK2), Collagen Type XIX Alpha 1 Chain (COL19A1), and Interleukin-6 Signal Transducer (IL6ST) are potential key molecules involved in the regulation of wound repair by hUCMSC-Exos.

**Discussion:**

In summary, hUCMSC-Exos regulate the functions of HUVEC and HSFs through miRNA, significantly promoting wound healing and tissue repair, suggesting that hUCMSC Exos therapy is a promising therapeutic approach.

## Introduction

The skin is the largest organ in the human body, serving as a barrier between the body and the external environment (Qiu et al., 2020). Burns, pressure ulcers, diabetes, and venous stasis can all lead to acute or chronic skin damages (Falanga, 2005; Yao and Eriksson, 2000). The skin has a remarkable repair and regeneration abilities. Epidermal wound healing is a dynamic process that is divided into four stages: hemostasis, inflammation, proliferation, and tissue remodelling (Reinke and Sorg, 2012; Guo and Dipietro, 2010; Maranda El Fau - Rodriguez-Menocal et al., 2017). The inflammatory phase is characterized by an influx of polymorphonuclear cells, followed by monocytes and macrophages. Which secrete growth factors and cytokines required for wound healing; Skin cells are stimulated by these growth factors, and healing progresses to the proliferative phase, including fibroplasia, matrix deposition, angiogenesis, and re-epithelialization. Remodelling is a dynamic phase during which various types of collagens continuously deposited and degraded (Singer and Clark, 1999; Ilan et al., 1998). The phases of wound healing must be precise, especially in the late stages of skin wound healing. Without proper wound management, the appearance and function of the skin can be affected by pathological scars (Singer and Clark, 1999; Karppinen et al., 2019).

Despite the self-repairing ability of the skin, wound management remains crucial for preventing infection and desiccation, alleviating pain, protecting the open wound, promoting healing, and preventing scarring, especially in cases of large-open wounds or burns (Nethi et al., 2019; Rowan et al., 2015). Wound healing is a complex and multifactorial physiological process. According to the type of wound and the method and approach of treatment, including swabbing for infection, cleaning tissue debris from the wound bed, transplantation, cell therapy, and application of wound dressings, the cost range of treatment is very wide. However, overall, the methods currently used to treat wounds are not ideal or 100% effective. Therefore, finding better and more cost-effective methods for treating skin wounds remain an urgent clinical need, and a challenging scientific and clinical issue.

Mesenchymal stem cells (MSCs) have been shown to promote epidermal wound healing. Firstly, MSCs can differentiate specifically in the microenvironments, such as into epidermal keratinocytes, endothelial cells, and pericytes, and transform into skin cells at sites of dermal injury, thereby inhibiting scar formation and promoting the repair of damaged tissue (Sasaki et al., 2008; Huo et al., 2018). Then, MSCs possess powerful immunomodulatory properties and can activate various cytoprotective genes in tissues (Uccelli et al., 2008; Brown et al., 2011; Francois et al., 2013; Aggarwal and Pittenger, 2005), and induce specific angiogenesis by secreting soluble factors, which is a key step in wound healing (Shabbir et al., 2015; Javazon et al., 2007; Kniazeva et al., 2011). In addition, MSCs can induce the polarization of macrophages from the proinflammatory M1 type to the anti-inflammatory M2 type, resulting in the production of interleukin-10 and further regulating immune response (Shi Y. et al., 2018; Zhang et al., 2020). Finally, the paracrine effect of MSCs is considered the main underlying mechanism in their ability to promote wound healing. Arno Annal et al. reported that umbilical cord mesenchymal stem cells (UCMSCs) increased the gene expression of transforming growth factor, hypoxia-inducible factor 1α, and plasminogen activator inhibitor-1, thereby accelerating the proliferation and migration of fibroblasts near the wound edge (Arno et al., 2014). Ma TSun j et al. reported that insulin growth factor 1, interleukin 6, transforming growth factor β, and other cytokines are enriched in the conditioned medium of adipose mesenchymal stem cells (ADMSCs) (Ma et al., 2017). These components play crucial roles in promoting epithelial and vascular regeneration during the proliferation stage. More and more studies have shown that MSCs can also promote wound healing by releasing exosomes containing mRNAs, microRNAs (miRNAs), and transcription factors that are crucial for the wound healing process (Shabbir et al., 2015; Kniazeva et al., 2011; Valadi et al., 2007; Aga et al., 2014), providing a promising therapeutic approach for wound healing.

Exosomes are nanosized extracellular vesicles (30–150 nm in diameter) produced from various types of cells and tissues, containing proteins, mRNAs, and miRNAs (Raposo et al., 2013; Colombo et al., 2014). Studies have demonstrated that MSC-derived exosomes have similar biological functions to those of MSCs and can serve as a possible therapeutic approach. The effects of exosomes from various types of cells on tissue repair have been extensively evaluated (Ren et al., 2019). Jiang et al. reported that human bone marrow-derived stem cell-derived exosomes (hBMSC-Exos) promote wound healing by inhibiting the TGF-β/Smad signalling pathway, reducing the expression of TGF-β1, promoting the secretion of TGF-β3 and decreasing the degree of scarring (Jiang et al., 2020). An Y et al. reported that ADMSC-derived exosomes (ADMSC-Exos) promote cell proliferation and migration in the early stage of wound healing after being absorbed by soft tissues, and increase the synthesis of type I collagen and type III collagen; in the late stage of healing, ADMSC-Exos inhibit scar growth by inhibiting collagen synthesis (An et al., 2021). Fang et al. demonstrated that hUCMSC-Exos enriched in specific miRNAs inhibit scar formation by inhibiting the TGF-β/Smad pathway (Li et al., 2016). MSC-Exos from different sources can promote the migration and proliferation of skin cells and the secretion of growth factors. Additionally, MSC-derived exosomes have been used as alternatives to MSCs in a variety of disease models, including neurological, cardiovascular, immune, renal, musculoskeletal, liver, respiratory, eye, and skin disease models, as well as cancer models (Nooshabadi et al., 2018).

So far, exosomes derived from MSCs have gathered increasing interest as a novel “cell-free” therapeutic strategy in wound healing and skin regeneration. However, most studies have focused on the use of ADMSC-Exos for the treatment of skin injury repair, with a tendency to evaluate the efficacy of exosome for the treatment endpoints. Unlike other hUCMSC-Exos, we selected hUCMSC-Exos for their non-invasively sourced abundant supply (Yu et al., 2023), low immunogenicity that lowers wound immune reaction risks (Hastuti et al., 2022), and multiple studies have also confirmed that they outperform ADMSC-Exos and hBMSC-Exos in terms of promoting angiogenesis (Zhou et al., 2023), fibroblast proliferation, and immune regulation (Heydari et al., 2025). These traits, validated to aid scarless wound healing, make them ideal for this study. Here, we investigated the therapeutic effects of hUCMSC-Exos on wound healing, elucidated the impact of exosomes on various stages of wound healing, provided a more detailed and comprehensive perspective on this intricate process, and identified the potential molecular mechanisms behind the function of extracellular vesicles. Our study provides experimental evidence and theoretical support for the prospective application of exosomes in wound healing.

## Materials and methods

### Isolation and culture of hUCMSCs

hUCMSCs were obtained and cultured according to previously described methods (Liao et al., 2023). Briefly, the umbilical cord was obtained from a healthy pregnant woman after informed consent was obtained. After the umbilical artery and umbilical vein were removed, the umbilical cord was rinsed three times with Dulbecco’s phosphate-buffered saline (D-PBS, Invitrogen), after which the soft gel tissues were cut into 0.5 cm × 0.5 cm pieces and added to gelatine-coated 10-cm tissue culture dishes. MSC NutriStem^®^ XF Basal Medium with MSC NutriStem^®^ XF Supplement Mix (Biological Industries) supplemented with 1% human platelet lysate (Sexton) was added to the culture, which was incubated at 37 °C until the hUCMSCs reached 80% confluency. The tissues were subsequently removed from the dishes, and the hUCMSCs were collected. hUCMSCs were derived from single donors. Passage 4 (P4) hUC-MSCs were used in all the experiments, and all the cells were cultured in complete medium (Biological Industries) supplemented with 1% human platelet lysate (Sexton).

### Culture of HUVECs and HSFs

Human umbilical vein endothelial cells (HUVECs) were purchased from the China Center for Type Culture Collection (Wuhan, China), and human skin fibroblasts (HSFs) were purchased from the American Type Culture Collection (ATCC, United States). HUVECs were cultured in endothelial cell medium (ScienCell, United States) supplemented with 5% fetal bovine serum (FBS) (HyClone), 1% endothelial cell growth factor (ECGS), and 1% penicillin‒streptomycin (Gibco). HSFs were cultured in high-glucose Dulbecco’s modified Eagle’s medium (DMEM; Gibco) supplemented with 10% FBS and 0.5% penicillin‒streptomycin. All the cells were cultured at 37 °C in a humidified atmosphere of 5% CO_2_.

### Characterization of hUCMSCs

The phenotypes of the hUCMSCs were analysed via flow cytometry after incubation with CD90-FITC, CD29-PE, CD105-APC, CD73-PE, CD45-FITC, CD34-PE, CD105-APC, CD14-FITC, HLA-DR-PerCP, and the corresponding isotype control antibodies. All the antibodies were purchased from BD Pharmingen (San Diego, CA, United States). hUCMSCs were analysed via a flow cytometer (BD FACS Calibur™, United States). To analyse the adipogenic, osteogenic, and chondrogenic differentiation of hUCMSCs, the cells were cultured in different appropriate differentiation media (Cyagen Biosciences Inc., Guangzhou, China) for 2–3 weeks, after which they were stained with Alizarin Red, Oil Red O, and toluidine blue, respectively.

### Isolation and identification of the hUCMSC-Exos

hUCMSCs were expanded from P3 to P4, and the P4 subculture supernatant was collected. Serum-free medium was used for culture during the experiment. Briefly, the culture media of the hUCMSCs were collected and centrifuged at 300 *g* for 10 min to remove the cells. The supernatant was transferred to a new centrifuge tube and centrifuged at 2,000 × g for 10 min. To remove dead cells and cell debris, the supernatant was transferred to another new centrifuge tube and centrifuged at 10,000 × g for 30 min. Next, the cell supernatant was ultracentrifuged at 100,000 × g for 90 min via a Beckman ultracentrifuge, and the supernatant was discarded and resuspended in precooled PBS. The exosomes were isolated by ultracentrifugation at 100,000 × g for 90 min, resuspended in the appropriate amount of PBS, and finally filtered into a new centrifuge tube with a 0.22 μm filter.

The protein content of the exosomes was quantified with a Pierce BCA protein assay kit (Thermo Scientific, United States). The surface characteristic protein markers of the exosomes, including CD9, CD81, CD63, TSG101, HSP70, Alix and Calnexin, were detected via Western blotting. All primary antibodies were purchased from Abcam and were detected by Western blotting. The morphology of the exosomes was detected with a Hitachi H-7650 transmission electron microscope (Tokyo, Japan), and the size distribution of the exosomes was measured via an NS300 instrument (Malvern Instruments Ltd., Malvern, United Kingdom) at 25 °C.

### hUCMSC-Exos labelling and internalization assay

Exosomes were labelled with a PKH26 kit according to the manufacturer’s instructions (Sigma, United States). For the internalization assay, the cells were seeded in 12-well plates at the proper density and treated with 10 μg of PKH26-labelled hUCMSC-Exos. The cells were incubated in an incubator with 5% CO_2_ for 24 h, washed twice with PBS and fixed in 4% paraformaldehyde for 10 min. Afterwards, the cell nucleus was stained with Hoechst (Solarbio, China), and the cytoskeleton was stained with FITC Phalloidin (Solarbio, China) according to the manufacturer’s instructions. The different fields of view were randomly imaged with laser confocal microscope (Zeiss, Germany), and statistical analysis was conducted.

### Cell proliferation, migration, and tube formation assays

For the cell proliferation assay, HUVECs and HSFs were trypsinized and then seeded at a density of 2 × 10^3^ cells/well in 96-well culture plates with 100 μL of the abovementioned specific cell culture medium. After overnight incubation, 1 μg (0.01 μg/μL), 5 μg (0.05 μg/μL), or 10 μg (0.1 μg/μL) of hUCMSC-Exos were added to each well after the culture medium was replaced, and proliferation was analysed according to the manufacturer’s instructions, with MTT (Sigma, Germany) added on days 1, 3, and 5, respectively.

For the cell migration assay, HSFs were seeded at a density of 1 × 10^5^ cells/well in 6-well plates and cultured to 90% confluence. Three parallel scratches were then made in each well with a 200 μL pipette tip, and the samples were washed with serum-free medium to remove the detached cells. The width of each scratch was measured as the baseline value, and 1 mL of medium was added to each well. The cells were stimulated with 10 μg (0.01 μg/μL) or 20 μg (0.02 μg/μL) of hUCMSC-Exos for 24 or 36 h. The scratch width was photographed with a microscope (Olympus, Tokyo, Japan), and the migration rate was immediately measured with ImageJ software.

For the tube formation assay, HUVECs (2 × 10^4^ cells per well) were seeded with 1 μg (0.01 μg/μL), 5 μg (0.05 μg/μL), or 10 μg (0.1 μg/μL) of hUCMSC-Exos or PBS in 96-well culture plates that had been coated with 60 μL of Matrigel Basement Membrane Matrix (Corning, United States), and 100 μL of medium was added to each well. Tube formation was detected via microscopy (Olympus, Tokyo, Japan) at 6 and 24 h of incubation, and the formation of tubular structures was analysed via ImageJ software.

### Apoptosis experiment

HUVECs and HSFs were seeded at a density of 3×10^5^ cells/well in 6-well culture plates, under normoxic (37 °C, 5% CO_2_, 21% O_2_) or hypoxic (37 °C, 5% CO_2_, 5% O_2_) conditions for 24 h. After which they were treated with 20 μg (0.02 μg/μL) of hUCMSC-Exos were cultured under normoxic conditions for 12 h, the cells were collected and stained with an Annexin V/7-AAD apoptosis kit (BD, USA), and the samples were analysed via a flow cytometer (BD FACS Calibur^TM^, USA)

### *In vivo* wound healing experiments in a mouse model

All animal experiments were approved by the ethics committee of the institutional review boards at Shenzhen Beike Biotechnology Co., Ltd. (No. BK-DWLL-2022-0001). This study adheres to the ARRIVE guideline 2.0 for the reporting of animal experiments.

All the mice were provided by Zhuhai BesTest Bio-Tech Co., Ltd. (Zhuhai, China). Fifty-four 8-week-old male BALB/c mice were divided into three groups: 1) the normal group (n = 18, not processed), 2) the saline group (n = 18, 200 μL of saline), and 3) the Exo-200 μg group (n = 18, 200 μg/200 μL of hUCMSC-Exos). The mice had unrestricted access to standard chow and drinking water and were were housed in a specific pathogen-free facility under a 12-h light/dark cycle. This experiment was performed at a constant temperature, and a bipolar electrocautery device with a 7-mm brass sheet was used as the tip. The temperature was set to 90 °C, and the weight of the tip (0.5 kg) was applied as the injury pressure to the mouse’s dorsal skin area for 5 s to obtain a scald wound of consistent area and severity. Within 24 h, Hematoxylin and Eosin (H&E) histopathological staining analysis was performed on the injured tissues of the mice to ensure that the back injury degree of each model mouse was consistent and that the injuries were deep second-degree burns (Supplementary Figure S6). The wounds were treated with subcutaneous injections of PBS or hUCMSC-Exos at five different locations, photographed on days 0, 4, 7, 10, 14, and 21 with cameras, and subsequently analysed with ImageJ software. Animals were euthanized via air displacement with 100% CO_2_, with a flow replacement rate of 30%–40% maintained throughout the process. This procedure was conducted on study days 4, 7, and 21, with six animals per group. Following the cessation of respiration, death was confirmed by cervical dislocation, after which tissue samples were harvested and processed.

### Quantitative real-time polymerase chain reaction (qRT‒PCR)

HUVECs and HSFs were seeded in 12-well culture plates, starved for 4 h, and then treated with 10 μg (0.01 μg/μL), 20 μg (0.02 μg/μL), or 30 μg (0.03 μg/μL) of hUCMSC-Exos or PBS for 24 h. Scar tissue samples were obtained from sacrificed mice at 4, 7, and 21 days after administration. Total RNA was isolated from cell lines or scar tissue samples with TRIzol reagent (Invitrogen, United States) following the manufacturer’s standard protocol. The RNA was reverse transcribed to cDNA via a reverse transcription kit (Takara, Dalian, China). Real-time PCR amplification was performed via a 7500 Real-Time PCR system (Applied Biosystems, United States). The expression of the target genes was assessed via the CT (2^−ΔΔCT^) method and normalized to that of GAPDH. The sequences of primers used in this study are shown in Table 1.

**TABLE 1:** Primers used for the amplification of human transcripts via real-time quantitative PCR.

Genes	Forward sequence (5′ to 3′)	Reverse sequence (5′ to 3′)
GAPDH	CAT​CAC​TGC​CAC​CCA​GAA​GAC​TG	ATG​CCA​GTG​AGC​TTC​CCG​TTC​AG
TNF-α	GGT​GCC​TAT​GTC​TCA​GCC​TCT​T	GCC​ATA​GAA​CTG​ATG​AGA​GGG​AG
IL-1β	TGG​ACC​TTC​CAG​GAT​GAG​GAC​A	GTT​CAT​CTC​GGA​GCC​TGT​AGT​G
MCP-1	GCT​ACA​AGA​GGA​TCA​CCA​GCA​G	GTC​TGG​ACC​CAT​TCC​TTC​TTG​G
VEGF	CTG​CTG​TAA​CGA​TGA​AGC​CCT​G	GCT​GTA​GGA​AGC​TCA​TCT​CTC​C
PDGF-α	CTG​GCT​CGA​AGT​CAG​ATC​CAC​A	GAC​TTG​TCT​CCA​AGG​CAT​CCT​C
PCNA	CAA​GTG​GAG​AGC​TTG​GCA​ATG​G	GCA​AAC​GTT​AGG​TGA​ACA​GGC​TC
α-SMA	TGC​TGA​CAG​AGG​CAC​CAC​TGA​A	CAG​TTG​TAC​GTC​CAG​AGG​CAT​AG
COL-I	CCT​CAG​GGT​ATT​GCT​GGA​CAA​C	CAG​AAG​GAC​CTT​GTT​TGC​CAG​G
COL-III	GAC​CAA​AAG​GTG​ATG​CTG​GAC​AG	CAA​GAC​CTC​GTG​CTC​CAG​TTA​G

### Enzyme-linked immunosorbent assay (ELISA)

Samples of mouse serum and wound tissue samples were collected 4, 7, and 21 days after the operation. The levels of IL-6, IL-17, IL-1β, TNF-α, and IFN-γ in scar tissues were measured separately via colorimetric sandwich ELISA kits (R&D Systems, United States). The absorbance at 450 nm was measured with a multimode microplate reader (Molecular Devices, United States). All the assays were performed following the manufacturer’s specific instructions.

### Histopathological analysis

Wound areas, including the surrounding skin, were collected at 4, 7, and 21 days after the operation. The samples were fixed with 4% paraformaldehyde (Phygene, China), dehydrated with a series of graded ethanol solutions, embedded in paraffin, and sliced into 5 μm-thick sections. Hematoxylin and eosin (H&E) staining and Masson’s trichrome staining were performed according to standard procedures. H&E staining was used to assess the infiltration of inflammatory cells during wound repair, whereas Masson’s trichrome staining was carried out to evaluate the content and maturity of the collagen in the wound beds. Photographs were taken at ×100 and ×200 magnification via a microscope and digital camera (Leica, Germany). Statistical analysis via ImageJ software was performed on five high-powered fields per sample.

### Immunohistochemistry analysis

For immunohistochemical staining, three animals from each group were examined. The sections were incubated with anti-TGF-β1, anti-CD31, anti-VEGF, anti-collagen I, and anti-collagen III antibodies (Abcam, 1:1000) overnight at 4 °C. The sections were subsequently rinsed with PBS three times and incubated with a secondary antibody solution for 1 h at room temperature. A microscope was used to obtain images, which were then analysed via ImageJ software. At least 5 fields (×400 magnification) were randomly selected for analysis; the data are presented as the percentage of positive cells out of the total number of cells.

### Bioinformatics analysis

Total small RNA constitution analysis for known/unknown RNA, miRNA Upset analysis, CPM gene expression density distribution, and correlation analysis of samples were conducted to verify the miRNA quality and consistent between three hUCMSC-Exos samples. We are used Gene Ontology (GO) and KEGG pathway enrichment analysis and annotation were performed by the cluster Profiler R package for top 22 high expressed miRNAs. TargetScan, miRanda, and miRNB online databases were applied for miRNA target-gene prediction. Cytoscape was used to construct the miRNA-mRNA network for predicting the target genes of miRNAs with high expression.

### Statistical analysis

Statistical analyses were conducted via GraphPad Prism 9.3.0 software (Version X, United States), and the results were compared via one-way analysis of variance (ANOVA). All quantitative data are representative of these experiments and are shown as the mean ± standard error of the mean (SEM). P < 0.05 was considered statistically significant.

## Results

### Identification of hUCMSCs and hUCMSC-Exos

hUCMSCs exhibited a typical fibroblast-like morphology when cultured *in vitro* (Supplementary Figure S1a). The cells also showed adipogenic, osteogenic, and chondrogenic differentiation capabilities under the appropriate conditions for 2–3 weeks after being stained with Oil Red O (for adipocytes), Alizarin Red S (for osteoblasts), and Alcian blue (for chondrocytes) (Supplementary Figures S1b–d). hUCMSCs were collected at P4 and subjected to flow cytometry for surface characterization. The results demonstrated that hUCMSCs were strongly positive for CD73, CD90, CD29, and CD105 but negative for CD79a, CD34, CD45, CD14, and HLA-DR (Supplementary Figure S1e).

**FIGURE 1:**
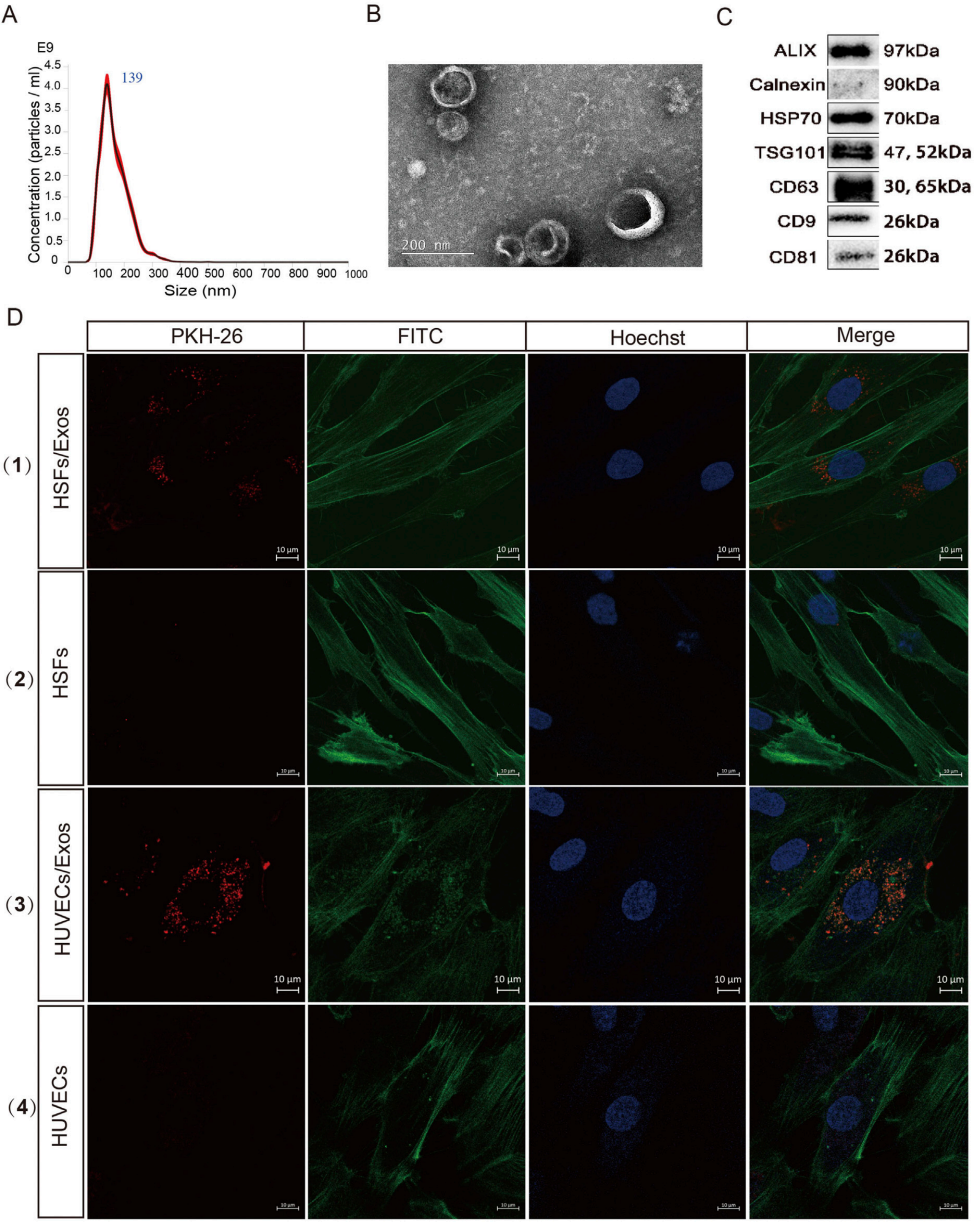
Characterization and internalization of the hUCMSC-Exos. **(A)** Diameter analysis of the hUCMSC-Exos via nanoparticle tracking analysis (NTA). **(B)** Transmission electron microscopy (TEM) was used to analyse the morphology of the hUCMSC-Exos. Scale bar, 200 nm. **(C)** Western blotting for the expression of CD9, CD81, CD63, TSG101, HSP70, Alix and Calnexin in hUCMSC-Exos (Full-length blots/gels are presented in Supplementary Material 1). **(D)** Figure Legends: **(D)** (1) (3) HUVECs and HSFs were incubated with PKH26-labelled Exos for 24 h, (2) (4) HUVECs and HSFs were incubated with PKH26 (without Exos) for 24 h, and fluorescence microscopy analysis was used to evaluate the internalization of the exosomes. Scale bar, 10 μm.

Exosomes were separated from the culture supernatant of hUCMSCs via ultracentrifugation. Nanoparticle tracking analysis (NTA), transmission electron microscopy (TEM), and Western blotting were performed to identify the diameter and concentration, morphological characteristics, and specific proteins of the hUCMSC-Exos, respectively. NTA was performed with a NanoSight instrument to measure the diameter of the exosomes, which had an average diameter of 139 ± 16.48 nm (Figure 1A). TEM revealed that the hUCMSC-Exos had a typical “saucer-like” exosome structure (Figure 1B). Moreover, the exosome markers were strongly positive for CD9, CD81, CD63, TSG101, HSP70, and Alix but negative for Calnexin, as determined by Western blotting (Figure 1C). Taken together, these results demonstrated that hUCMSC-derived exosomes were successfully isolated from the culture supernatant, which is similar to many previous reports (Zhou et al., 2021; Xiao et al., 2021).

### hUCMSC-Exos are internalized by HUVECs and HSFs

Exosomes can be internalized through several mechanisms, including fusion and endocytosis (Gonda et al., 2019; Montecalvo et al., 2012). To investigate whether the isolated exosomes could enter the cytoplasm of target cells, FITC Phalloidin-stained HUVECs and fibroblasts were incubated with PKH26-labelled Exos for 24 h. Both types of cells presented with red fluorescence in their cytoplasm, which suggested that the hUCMSC-Exos were readily internalized by HUVECs and HSFs (Figure 1D). The results showed that exosomes could be successfully delivered into target cells, which is the necessary process for performing their functions.

### hUCMSC-Exos promote cell proliferation, migration and angiogenesis *in vitro*

**FIGURE 2:**
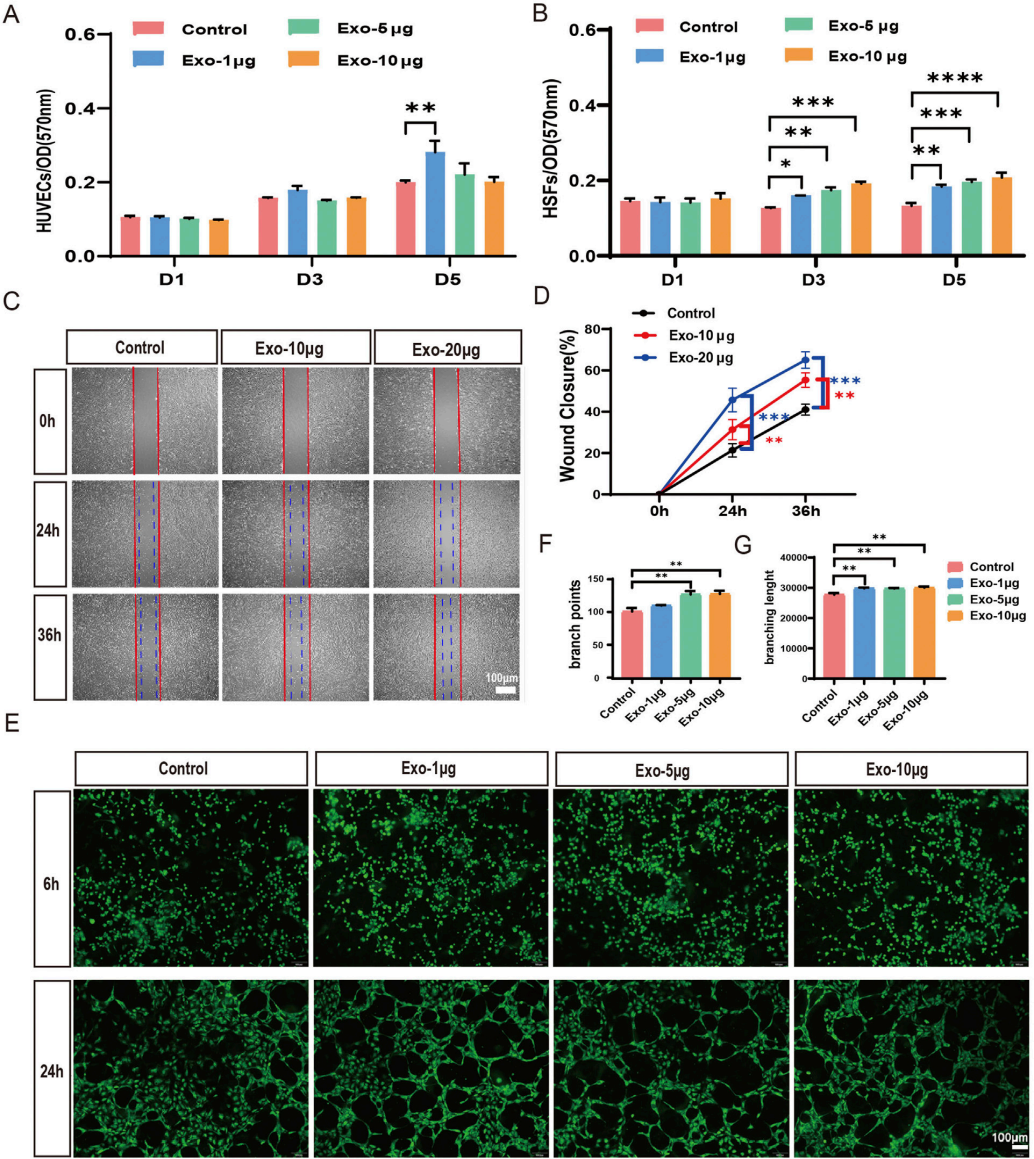
hUCMSC-Exos significantly induce the migration and proliferation of HSFs and enhance angiogenesis in HUVECs. **(A,B)** The proliferation of HUVECs **(A)** and HSFs **(B)** at days 1, 3, and 5 was analysed via the MTT assay after treatment with 1 μg, 5 μg, or 10 μg of hUCMSC-Exos; the control group was not subjected to Exos (n = 3). **(C)** Images of migrated HSFs were taken at 24 h and 36 h after treatment with PBS, 10 μg, or 20 μg of hUCMSC-Exos. Scale bar, 100 μm. **(D)** The percentages of wound closure were quantified at 0 h, 24 h and 36 h among the different treatment groups (n = 3). **(E)** Tube formation of HUVECs was evaluated at 6 h and 24 h after treatment with 1 μg, 5 μg, or 10 μg of hUCMSC-Exos. Scale bar, 100 μm. **(F,G)** Qualifications of tube branch points and branch lengths are shown (n = 3). *p < 0.05; **p < 0.01; ***p < 0.001; ****p < 0.0001.

We conducted a series of assays to investigate whether the hUCMSC-Exos could promote cell proliferation, migration and angiogenesis. The proliferation of HUVECs and HSFs cultured with hUMSC-Exos for 1, 3, or 5 days. We found that hUCMSC-Exos significantly promoted HSF proliferation in a dose-dependent manner at days 3and 5; however, they did not have an obvious influence on HUVEC proliferation at different time points. Only the Exo-1 μg group on day 5 promoted proliferation compared with the control group (Figures 2A,B). As shown in Figures 2C,D, hUCMSC-Exos effectively enhanced cell migration in a dose-dependent manner compared with that in control cells, with migration increasing 1.5-fold and 2.3-fold in 10 μg and 20 μg hUCMSC-Exos groups, respectively. Moreover, an *in vitro* tube formation assay was conducted to evaluate the ability of the hUCMSC-Exos to promote the formation of tubular structures in endothelial cells, and the results revealed that the hUCMSC-Exos significantly promoted tube formation after 24 h of culture (Figures 2E–G). Together, these results indicate that hUCMSC-Exo treatment promotes the proliferation and migration of HSFs and enhances angiogenesis in HUVECs.

### hUCMSC-Exos decreased cell apoptosis and upregulated expression of regenerative genes

To determine whether hUCMSC-Exos affect the apoptosis of cells, we established apoptosis models in which HUVECs and HSFs were induced with Hypoxia and then incubated with exosomes. We conducted flow cytometry analyses via Annexin V/7-AAD double-staining. As shown in Supplementary Figures S2a,b, compared with that of the Hypoxia group, the apoptosis rate of the cells treated with the hUCMSC-Exos was significantly lower, suggesting that the hUCMSC-Exos inhibited the apoptosis of the HUVECs (Supplementary Figure S2a) and HSFs (Supplementary Figure S2b) *in vitro*. Given the positive results that we obtained in the apoptosis study, we further examined the effects of hUCMSC-Exos on the regenerative gene regulation of cells. Our results revealed that exosomes increased the expression of key factors, such as PCNA, MCP-1, TGF-β, PDGFα, PEGF, VEGF, EGF, and FGF2, in target cells; among these factors, exosomes regulate MCP-1 (4 fold)in endothelial cells most obviously, and MCP-1 can promote the chemotaxis of inflammatory cells and the proliferation and migration of vascular endothelial cells. However, the PDGF-α (2.8 fold) and FGF (2.2fold) genes of fibroblasts are significantly regulated and are involved mainly in cell proliferation and differentiation and the induction of vascular hyperplasia. These results suggest that exosomes play a significant role in regulating the expression of genes associated with tissue repair and regeneration (Supplementary Figures S2c,d).

### hUCMSC-Exos promote cutaneous wound healing

**FIGURE 3:**
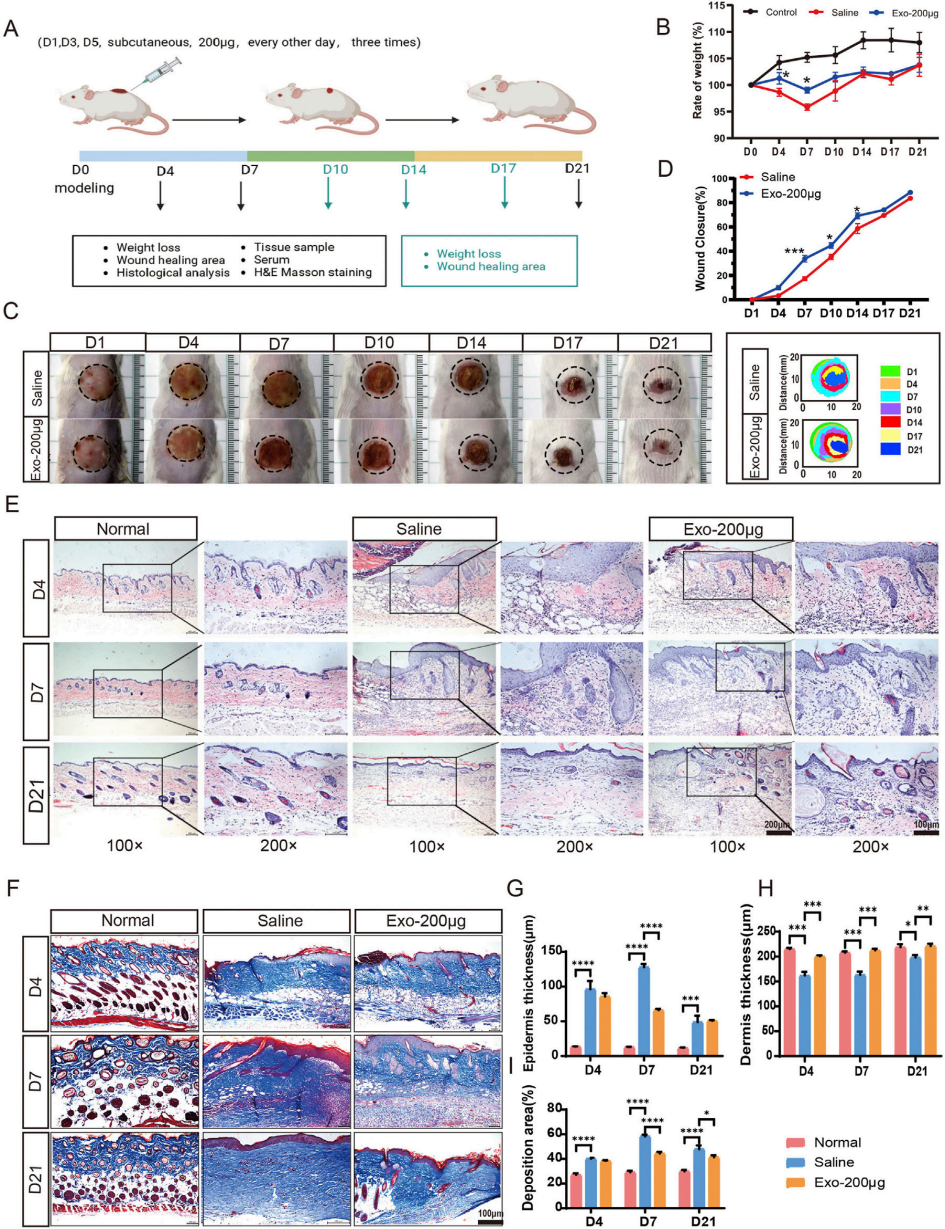
hUCMSC-Exos promoted cutaneous wound healing in a mouse model. **(A)** Representation of the experimental procedure *in vivo*. The mice were randomly divided into three groups (n = 18/group): the normal group, saline group, and Exos group (200 μg exos/mouse). The hUCMSC-Exos or saline were applied via local injection around the wound area and were injected three times in total, 1 day apart (day 1, day 3, and day 5). The experimental observation points were set at days 4, 7, and 21, and equal numbers of mice were sacrificed in each group (n = 6/group). **(B)** Body weight changes in the mice during the treatment phase. **(C)** Representative images of the healing process of wounds in the saline- and hUCMSC-Exo-treated groups at different time points are shown, along with the corresponding wound closure trajectories over 21 days. **(D)** The wound closure rates of both groups at different time points were calculated via ImageJ software. **(E,G,H)** Representative images of wounded skin sections from different groups were evaluated by H&E staining at days 4, 7, and 21 **(E)**, and the quantification data of epidermis thickness **(G)** and dermis thickness **(H)** were calculated; scale bar = 200 μm in the 100× images (left panel); scale bar = 100 μm in the 200× images (right panel). **(F,I)** Representative images of fibrosis in all groups at days 4, 7, and 21 are shown via Masson’s trichrome staining **(F)**, and the collagen deposition area was quantified **(I)** scale bar, 100 μm. Mean ± SEM. *P < 0.05; **P < 0.01, ***P < 0.001; ****p < 0.0001.

To evaluate the therapeutic potential of the hUCMSC-Exos in terms of the wound healing rate and scar formation, a mouse wound model with scalded skin was used. Our model produces a deep partial-thickness burn (deep second-degree), where signs of dermal injury included vascular damage or blockage, collagen damage, and dermal appendage damage but does not involve the complete destruction of skin appendages, making it a suitable model for studying repair processes (Supplementary Figure S6). Cutaneous wounds were created on the dorsal skin of male BALB/c mice, followed by local injection of 200 μg of hUCMSC-Exos (n = 18/group) or an equal volume of diluent (saline) around the wound area once daily for three consecutive days (Figure 3A). The body weight changes of the mice during the modelling and treatment phases were documented, and the Exos group at days 4 and 7 presented greater body weights than did the saline group and significantly differed from the saline group in terms of wound healing (Figure 3B). As shown in Figures 3C,D, cutaneous wound closure was greater in the Exos group than in the saline group after the subcutaneous administration of the hUCMSC-Exos. Notably, there was a significant difference between the two groups, especially on day 4.

During the wound healing process, hUCMSC-Exos may stimulate nearby stem cells to proliferate, differentiate and repair tissue through paracrine signalling (Zhou et al., 2023). Moreover, reepithelialization and angiogenesis occur (Liu et al., 2021). As shown by the H&E staining results in Figure 3E, the histological structure of the regenerated dermis showed that the structure of the normal mouse dermis and epidermis was regular; dermal cell destruction, epidermal thickening, and hyperkeratosis were observed in the saline group at days 4 and 7; on the other hand, the dermal cell structure was noticeably more regular in the Exos group than in the saline group, and there was a significant improvement in stratum corneum hyperkeratosis. On day 21, skin injury repair was assessed in both groups of mice, and better results were observed in the Exos group (Figures 3G,H). The Masson staining results revealed skin fibrosis: the Exos group presented normal collagen deposition and more skin appendages, whereas the saline group presented excessive collagen deposition at days 7 and 14 (Figures 3F,I). Similarly, we detected high expression of the PCNA and α-SMA genes at the site of the injured skin, which are highly correlated with cell proliferation and the degree of fibroblast-to-myofibroblast transformation and wound contraction (Supplementary Figures S4c,d). Taken together, these results indicate that exosomes strongly promote wound repair and skin regeneration.

### hUCMSC-Exos significantly promote angiogenesis

**FIGURE 4:**
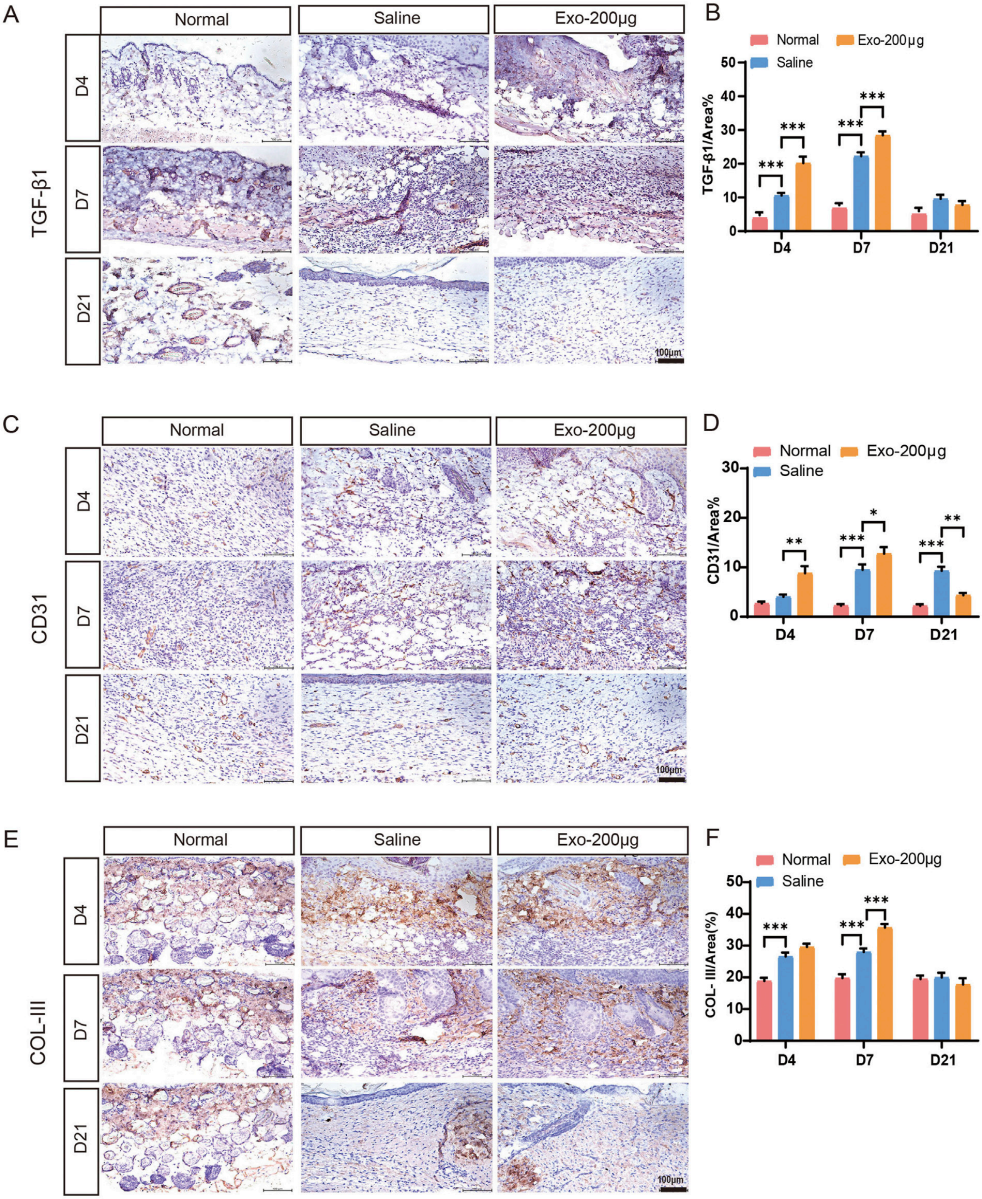
Immunohistochemical analysis of angiogenesis and collagen after wound healing in different groups on days 4, 7, and 21. **(A–F)** Immunohistochemical staining and quantitative analysis of TGF-β1, CD31, and collagen III expression in skin wound tissues. The results are presented as the mean ± SEM; n = 6 for each group. Scale bar, 100 μm *p < 0.05, **p < 0.01, ***p < 0.001.

Angiogenesis is an essential physiological process for maintaining provisional granulation tissue. Our experiments revealed that hUCMSC-Exos effectively promoted angiogenesis in a wound mouse model *in vivo* (Figures 4A–D; Supplementary Figures S3a,b). Immunostaining for TGF-β1 (Figure 4A), CD31 (Figure 4C) and VEGF (Supplementary Figure S3a) in pathological skin tissues was performed at days 4, 7, and 21 after treatment with the hUCMSC-Exos to investigate their proangiogenic effects *in vivo*. Compared with the saline group, the Exos group presented notable increases in the expression of TGF-β1-, VEGF- and CD31-positive microvessels in skin tissue at days 4 and 7 (Figures 4B,D; Supplementary Figure S3b), which indicates that Exos significantly stimulate angiogenesis. However, the expression of CD31 and VEGF in the Exos group had already decreased on day 21 (Figure 4D; Supplementary Figure S3b). This finding indicated a tendency toward normalization at an earlier process stage, and the Exos group underwent skin remodelling at a more accelerated rate. These findings closely resemble the typical angiogenic patterns observed in the skin of wounded mice (Supplementary Figure S3e). In addition, we detected the expression of the VEGF and PDGF genes, which are highly correlated with angiogenesis, in injured tissues and reached similar conclusions (Supplementary Figures S4a,b). Specifically, the Exos group exhibited a more robust angiogenic response in the injured skin, characterized by earlier onset, increased vessel numbers, and higher vessel densities. In contrast, the saline group exhibited a less pronounced angiogenic response, with fewer vessels and a greater amount of inflammatory exudate. These results indicate that hUCMSC-derived exosomes have superior proangiogenic properties.

### hUCMSC-Exos significantly promote collagen synthesis

Additionally, we conducted experiments to determine the potential impact of hUCMSC-Exos on collagen synthesis at the wound site. As determined by Masson’s trichrome staining, wounds treated with hUCMSC-Exos exhibited greater collagen maturity than those in the other groups did (Figures 3F,I). To examine the influence of the hUCMSC-Exos on collagen, IHC staining was performed to detect the expression of collagen I and collagen III (Figures 4E,F; Supplementary Figures S3c–e). This analysis revealed that the hUCMSC-Exos had a stimulatory effect on collagen deposition, which is consistent with previous reports (Lin et al., 2024). Notably, increased collagen maturity was observed in the wound area, accompanied by a reduced ratio of collagen III to collagen I, which became more apparent with repeated hUCMSC-Exos treatments than in the saline groups. In essence, the wound healing process involves a pivotal transition in collagen types; type III collagen is initially predominant but is subsequently gradually replaced by type I collagen (Gelse et al., 2003). Furthermore, we obtained similar results by testing the gene expression of collagen III to collagen I in the injured tissues (Supplementary Figures S4e–g). Thus, these results demonstrated that hUCMSC-Exos regulate collagen deposition and organization, promote skin repair.

### hUCMSC-Exos significantly suppress wound inflammation

**FIGURE 5:**
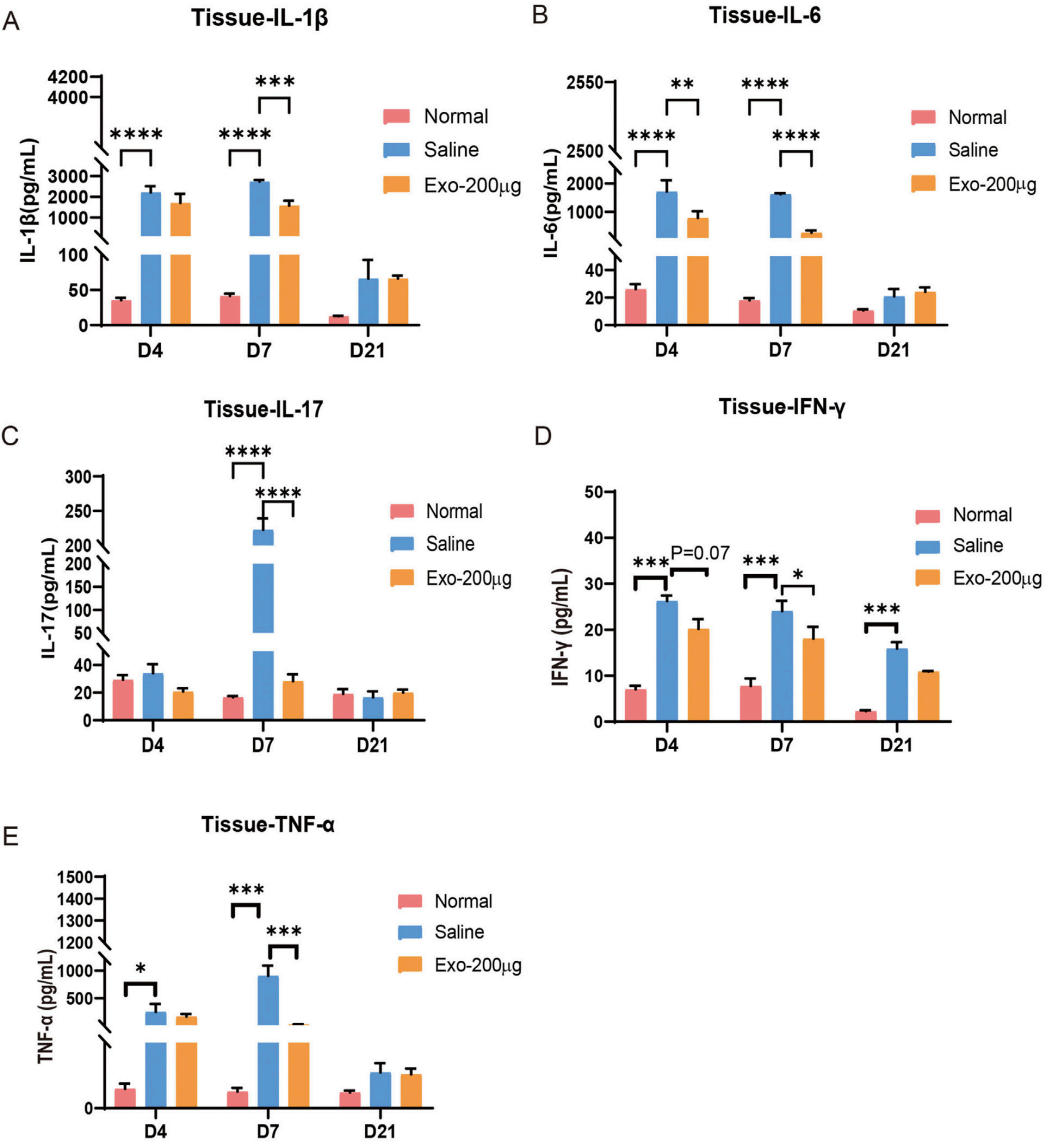
hUCMSC-Exos obviously reduced the expression of inflammatory factors in the wounded skin tissue of mice. **(A–E)** The levels of IL-1β **(A)**, IL-6 **(B)**, IL-17 **(C)**, IFN-γ **(D)**, and TNF-α **(E)** in the tissues of the mice were detected via ELISA. The results are presented as the mean ± SEM; n = 6 for each group. *p < 0.05; **p < 0.01; ***p < 0.001, ****p < 0.0001.

Our study investigated the impact of hUCMSC-Exos on wound inflammation in mice. To evaluate the anti-inflammatory potential of these exosomes, we employed ELISA to quantify the expression levels of inflammatory markers, such as IL-1β, IL-17, IL-6, TNF-α, and IFN-γ, in tissue homogenates from mice treated with exosomes or saline. Our findings revealed that inflammation occurred during the early stages of wound healing (Figures 5A–E). Compared with that in the normal group, the expression of inflammatory factors in the saline-treated group significantly increased. Importantly, we observed a significant reduction in the expression of these inflammatory markers after hUCMSC-Exos treatment, especially at the 7-day time point. Similarly, we found that the expression of inflammatory cytokines in mouse serum samples was comparable (Supplementary Figures S5a–d). These results collectively indicate that hUCMSC-Exos possess robust anti-inflammatory properties, thus accelerating the skin repair process.

### miRNA sequencing and bioinformatics analysis of the hUCMSC-derived exosomes

**FIGURE 6:**
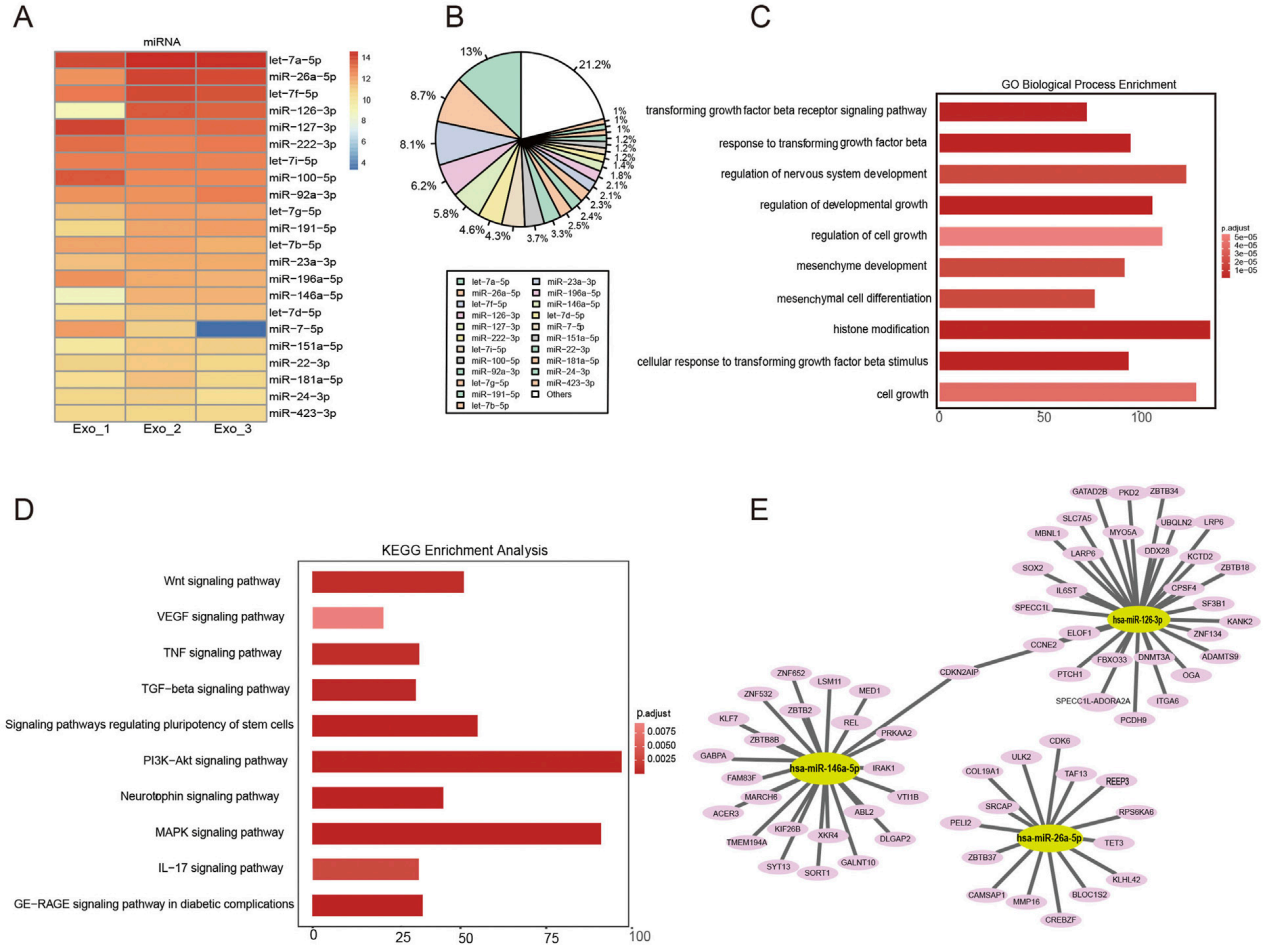
Functional analysis of hUCMSC-Exo-derived miRNAs. **(A)** The top 22 most highly expressed miRNAs in hUCMSC-Exos are shown. **(B)** The percentages of the top 22 miRNAs in hUCMSC-Exos were calculated. **(C)** GO analysis revealed that the miRNA‒target genes were involved in histone modification, the regulation of developmental growth, and the response to transforming growth factor beta. **(D)** KEGG pathway analysis revealed that the miRNA target genes were involved in the PI3K/Akt, MAPK, and Wnt signalling pathways. **(E)** The miRNA‒mRNA interaction network showing the predicted target genes involved in the immune regulation of the miRNAs in hUCMSC‒Exos.

Exosomes can posttranscriptionally regulate coding genes through miRNAs, thereby affecting the biological activities of recipient cells (Li B. et al., 2021; Li C. et al., 2021). To explore the potential molecular mechanisms of the hUCMSC-Exos RNA cargo in wound healing, the small RNAs from the hUCMSC-Exos and their target genes were examined via RNA sequencing. We observed a significant correlation in the enrichment patterns of miRNA molecules across the three batches of exosomes, indicating a high degree of consistency and reproducibility in our findings (Figure 6A). Moreover, let-7a-5p, miR-26a-5p, let-7f-5p, miR-126-3p, miR-127-3p, miR-222-3p, let-7i-5p, miR-100-5p, and miR-92a-3p were highly expressed in the hUCMSC-Exos (Figure 6B). Interestingly, the high levels of several specific miRNAs, such as miR-26a-5p, miR-126-3p, miR-146a-5p, and miR-127-3p, may suggest a potential inhibitory effect of hUCMSC-Exos on wound healing.

Additionally, functional enrichment analysis revealed the dominant pathway enriched in the hUCMSC-Exos. We performed a functional enrichment analysis of 22 selected miRNAs via Gene Ontology (GO) and Kyoto Encyclopedia of Genes and Genomes (KEGG) enrichment analyses. GO analysis of the selected miRNAs revealed similar affected processes, such as the cell cycle, cell motility and differentiation (Figure 6C). KEGG pathway enrichment analysis revealed that the miRNAs enriched in the PI3K‒Akt signalling pathway, MAPK signalling pathway, and Wnt signalling pathway (Figure 6D). The interactions of known miRNAs and mRNAs with respect to cell proliferation and migration, signalling molecules and interactions, signal transduction, and the immune system were observed via Cytoscape (Figure 6E). The most abundant miRNAs, such as miR-126-3P, miR-26-5P, and miR-146a-5P, were found to target genes encoding ULK2, COL19A1, IL6ST, SLC7A5, CDKN2AIP, MED1, KLF7, GABPA, and IRAK1, which are associated with skin injury repair. Notably, these results are consistent with the finding that the most common miRNAs in hUCMSC-Exos and their target genes promote cell proliferation and reduce inflammatory responses. Together, these bioinformatic results suggest that exosome-derived miRNAs can contribute to promoting skin repair and regeneration.

## Discussion

Skin wound healing is a well-organized physiological process that involves the cooperation of various cell types and their derivative molecules (Reinke and Sorg, 2012; Gantwerker and Hom, 2011). Many studies have reported that the implantation of exogenous MSCs can promote the repair of skin wounds. MSC therapy (Athanerey et al., 2017; Tsai et al., 2018; Khademi et al., 2020)promotes skin repair and rejuvenation through paracrine actions, immunological regulation, inflammation management, and cell differentiation (Li et al., 2024). Many previous works have revealed that the paracrine actions of MSCs underlie their biological functions and that their secretion of exosomes and microvesicles is primarily responsible for their therapeutic properties (Tsiapalis and O'Driscoll, 2020). MSC-derived exosomes have an extensive array of potential applications, including cancer treatment, wound healing, tissue regeneration, and numerous other critical domains (Hong et al., 2019). In contrast to hUCMSC-mediated therapy, hUCMSC-Exos mimic the function of host cells and can effectively mitigate the risk of tumor development, immune-induced rejection, limited cell survival, loss of function, or senescence-triggered genetic instability.

MSC-Exos are effective therapeutic agents that promote the wound-healing process. This effectiveness is attributed to their ability to activate signalling pathways, which are associated with the promotion of cell migration, vascularization, collagen deposition, and the inflammatory response (Nooshabadi et al., 2020; Rosca et al., 2018). In this study, we first evaluated the effects of hUCMSC-Exos on fibroblasts and endothelial cells, the key cell types involved in injured skin repair. Moreover, we investigated the therapeutic effects of hUCMSC-Exos in a mouse model of wound healing and preliminarily predicted the possible underlying mechanisms by which hUCMSC-Exos exert prorepair effects. Our *in vitro* results demonstrated that hUCMSC-Exos effectively enhanced the proliferation and migration of HSFs in a dose-dependent manner. Additionally, hUCMSC-Exos also significantly promoted the proliferation and tube formation of HUVECs, which is consistent with Zhang B’s studies (Zhang et al., 2015a; Zhang et al., 2015b). In addition, we found that hUCMSC-Exos have antiapoptotic properties and can promote the expression of related growth factors in fibroblasts and endothelial cells (Supplementary Figure S2). Moreover, our study revealed that hUCMSC-Exos could be easily endocytosed by HUVECs and fibroblasts, which suggests that hUCMSC-Exos can act as appropriate vehicles for the transport of various biomolecules and signals to these cells. These experimental results provide strong evidence that exosomes promote skin injury repair.

The process of skin regeneration typically involves four overlapping phases: the inflammation stage, the angiogenesis stage, the proliferation stage (which consists of cell proliferation and re-epithelialization), and the final remodelling stage (Stephens and Thomas, 2002; Rodrigues et al., 2019). Inflammation constitutes the initial response in typical wound repair. However, excessive inflammation results in delayed or even failed wound healing (Broughton et al., 2006). In our *in vivo* study, we observed skin wound healing in mice throughout the repair phase at days 0, 7, 14 and 21. In contrast, previous studies have focused more on the validation of the effect of wound healing in the later stages. Our *in vivo* experiments revealed that in the acute inflammatory phase (Day 4), IL-1β, IL-6, TNF-α, and IFN-γ rose rapidly: the model group showed high local expression of these factors, while exosomes reduced the excessive activation of anti-inflammatory factors in the model group, and serum pro-inflammatory factors were lower than those in skin due to effective local control. El Daly et al. (Eldaly et al., 2022) revealed that MSC-derived exosomes to reduced serum IL-1β, IL-6, and TNF-α via M1-to-M2 macrophage polarization, and Weber et al. (Weber et al., 2023) noted serum exosomal IL-6 peaked at 50%–60% of skin levels with a 1–2-day lag, consistent with this study. Moving to the proliferative phase (Day 7), moderate IL-17 peaked in the model group, while the exosome group lowered this IL-17 peak and maintained anti-inflammatory effects, with serum factors peaking (higher than Day 4). Wang et al. (Wang et al., 2022) showed adipose MSC exosomes reduced skin IL-17A by inhibiting Th17 differentiation and upregulating Treg cells. By the remodeling phase (Day 21), wounds were basically healed, local inflammation returned to homeostasis, and both groups had low, non-tissue-damaging levels of pro-inflammatory factors. Similarly, we observed the same phenomenon in the injured skin of the mice (Supplementary Figure S3). These data suggest that hUCMSC-Exos can reduce inflammation and accelerate the wound healing process, which is particularly important during the proliferation stage, neoangiogenesis, collagen deposition, re-epithelialization, and wound contraction.

During the proliferative stage, the wound healing process is initiated through the formation of provisional granulation tissue and the proliferation of cells in the affected area. The proliferation of fibroblasts and the formation of the extracellular matrix (ECM) provide the material basis for wound repair. Neovascularization is a key step in promoting wound repair; the greater the density of blood vessels is, the greater the ability to transport nutrients and oxygen to promote skin cell migration and proliferation (Addis et al., 2020). Collagen type III is the predominant collagen type in healing wounds, and it serves as the major component of granulation tissue (Singh et al., 2023). In addition, extensive angiogenesis occurs at this stage (Broughton et al., 2006). In our *in vitro* study, we found that hUCMSC-Exos promoted the proliferation and migration of HSFs and enhanced the proliferation and tube-forming ability of HUVECs. These results are consistent with those of previous studies (Sun et al., 2017). In our *in vivo* study, H&E staining results showed that in the model group, the vascular-related indicators (CD31/VEGF) and TGF-β1 presented a synergistic upward trend within 7 days, due to the interventional effect of exosomes, accelerated all stages of wound healing, resulting in more rapid and sufficient changes in the expression of each indicator. Moreover, we also observed that greater microvessel density on the back skin of mice in the Exos-treatment group (Supplementary Figure S3). In addition, given that angiogenesis provides a route for the transportation of cells and biomolecules, the enhanced vascularization observed here may also promote the targeted delivery of exosomes or the recruitment of endogenous cells to the injury site, and this mechanism deserves in-depth exploration in subsequent studies (Pecoraro et al., 2021; Moeinabadi-Bidgoli et al., 2023). The expression timing of collagen III was highly consistent with that of TGF-β1, reflecting the regulatory effect of TGF-β1 on early collagen synthesis, we found that the Exos-treatment group presented greater collagen deposition, as evidenced by Masson’s trichrome staining, and more prominent immunostaining of COL III at both days 4 and 7, the COL III/COL I ratio indicated that both the production of COL III and its conversion to COL I in the Exos-treatment group occurred earlier than in the model group. The high expression of collagen I on day 21 and the decrease in TGF-β1 showed a reverse trend, which reflected the better collagen remodeling efficiency of the Exos-treatment group, thereby promoting faster and better wound healing. The exosomes significantly promoted wound healing and tissue regeneration and reduced the inflammatory response. These efforts may be attributed to the fact that exosomes contain or promote the secretion of large amounts of growth factors, such as VEGF, bFGF and TGF-β1, which have been shown to support the angiogenic potential of cells (Cooper et al., 2018). Our results revealed that these exosomes significantly increased the expression of key factors, such as PCNA, MCP-1, TGF-β, PDGFα, PEGF, VEGF, EGF, and FGF2, in target cells (Supplementary Figures S2c,d). These factors are important for regulating the intricate processes of skin repair and regeneration. In general, our findings provide valuable insights into the potential therapeutic applications of MSC-Exos in promoting skin healing and regeneration.

MSC-derived exosomes contain proteins, mRNAs, and miRNAs that function in a variety of biological processes (Ramakrishnaiah et al., 2013; Cheng et al., 2014; Kordelas et al., 2014). The main functional RNA component in exosomes is miRNA, which can be efficiently transmitted to other cells and exert diverse effects through exosome integration (Alexander et al., 2015). We also sought to clearly distinguish the mechanisms by which exosomal miRNAs may be involved in the regulation of wound healing. miRNA sequencing revealed that the majority of the 22 most highly expressed miRNAs in the hUCMSC-Exos were associated with stimulating cell proliferation and migration and attenuating inflammatory responses. Functional enrichment analysis of the miRNAs revealed several enriched biological processes and signalling pathways. We found that the miRNA target genes of the hUCMSC-Exos were enriched in homeostatic processes; cell differentiation; and the PI3K/AKT, MAPK, Wnt and TGF-β signalling pathways. These pathways have been the subject of many previous studies. Zhang et al. reported that exosomes from adipose-derived stromal stem cells (ASCs) promote fibroblast proliferation and collagen secretion via the PI3K/AKT signalling pathway (Zhang et al., 2018). Human umbilical cord MSC-derived exosomes can also increase HaCaT cell proliferation and migration through activation of the WNT/β-catenin signalling pathway (Zhang et al., 2015a). Numerous studies have been conducted on miRNA molecules in skin injury models (Shi J. et al., 2018; Li et al., 2015; Aunin et al., 2017). However, we identified four molecules, namely, miR-26a-5p, miR-126-3p, miR-146a-5p and miR-127-3p, that have not been extensively studied in the context of skin repair, despite their relevance in the regulation of biological processes. Through further target gene prediction, we identified ULK2, COL19A1, and IL6ST as potential novel molecules of hUCMSC-Exos that may play a role in regulating the damage repair process. Unc-51-like autophagy activating kinase 2 (ULK2) is a crucial kinase in the autophagy process that is involved in regulating the initiation of cellular autophagy. During the process of injury repair, cellular autophagy is essential for the removal of damaged organelles and proteins, as well as for promoting cell survival and regeneration (Debnath et al., 2023). COL19A1 is a member of the collagen family, and collagen is a major component of the ECM. The ECM is essential for maintaining the structural integrity of tissues and for promoting cellular attachment (Ricard-Blum and Ruggiero, 2005). Interleukin-6 (IL-6) plays a pivotal role in the inflammatory response and immune system activation. Its production can be triggered by the activation of downstream signalling pathways, such as Janus kinase (JAK)/signal transducer and activator of transcription (STAT) pathways. IL6ST has been demonstrated to facilitate cell proliferation, differentiation and migration, thereby accelerating the repair of damaged tissue (Jones and Jenkins, 2018). The role of these target genes in the exosome-mediated repair of skin damage has yet to be fully elucidated. These findings may provide a basis for further investigations into the underlying molecular mechanisms involved.

## Conclusion

In brief, we identified a critical role for hUMSC-Exos in wound healing, mainly through their ability to modulate inflammatory responses, stimulate vascularization, and promote ECM formation and collagen synthesis to enhance the recovery of the skin barrier at the site of injury. However, this study has several limitations. We focused primarily on small RNA carriers within exosomes and predicted their involvement in pathways and potential target genes via bioinformatic analysis. While we have not yet conducted validation to pinpoint a specific miRNA that plays a dominant regulatory role, this remains a pivotal aspect of our future research. Nonetheless, our efficacy verification data from *in vitro* and *in vivo* studies are remarkably thorough and comprehensive. For the animal experiments, we measured healing indicators at multiple time points to monitor the changes in the characteristics of the wound healing process. These comprehensive data are highly important for determining treatment regimens for the future clinical application of exosomes.

## Data Availability

The datasets presented in this study can be found in online repositories. The names of the repository/repositories and accession number(s) can be found below: https://www.ncbi.nlm.nih.gov/, PRJNA1164939.

## Abbreviations

IL-6: Interleukin-6; ULK2: Unc-51-like autophagy activating kinase 2; COL19A1: Collagen Type XIX Alpha 1 Chain; IL6ST: Interleukin-6 Signal Transducer. hUCMSC-Exos, human umbilical cord mesenchymal stem cell-derived exosomes; NTA, nanoparticle tracking analysis; WB, Western blotting; TEM, transmission electron microscopy; HSFs, human skin fibroblasts; HUVECs, human umbilical vein endothelial cells; ADMSCs, adipose mesenchymal stem cells; hBMSC-Exos, human bone marrow-derived stem cell-derived exosomes; D-PBS, Dulbecco’s phosphate-buffered saline; P4, passage 4; H&E, hematoxylin and eosin; RT‒PCR, quantitative reverse-transcriptase polymerase chain reaction; FGF2, fibroblast growth factor 2; PCNA, proliferating cell nuclear antigen; TGF-β1, transforming growth factor beta 1; VEGF, vascular endothelial growth factor A; α-SMA, α-smooth muscle actin, aorta; MCP-1, monocyte chemoattractant protein-1; PDGFα, platelet derived growth factor alpha; PEGF, pigment epithelium-derived factor; VEGF, vascular endothelial growth factor; EGF, epidermal growth factor.
