# Supplementary material for: Umbilical cord mesenchymal stem cell-derived exosomes promote wound healing and skin regeneration via the regulation of inflammation and angiogenesis
Source: Front Bioeng Biotechnol. 2025 Nov 3;13:1641709. doi: 10.3389/fbioe.2025.1641709 (PMC12620388; doi:10.3389/fbioe.2025.1641709)
Supplement: Supplementary file 1 [file DataSheet1.pdf]

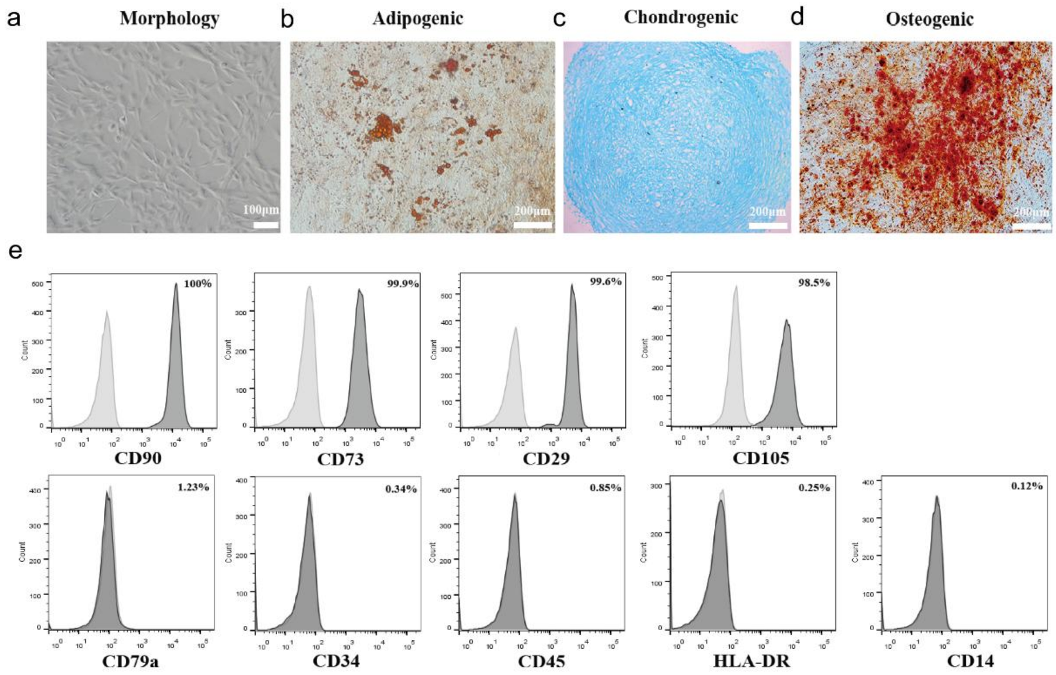

Figure S1. Characterization of hUCMSCs. (a) hUCMSCs were adherent and fibroblast-like cells. (b) Adipogenic differentiation assay showed that hUCMSCs could differentiate into adipocytes as stained with Oil Red O. (c) Chondrogenic differentiation assay showed that hUCMSCs could differentiate into chondrogenic as evidenced by Alcian Blue staining. (d) Osteogenic differentiation assay showed that hUCMSCs could differentiate into osteocytes as evidenced by Alizarin Red staining. (e) Flow cytometry revealed that more than 95% of hUCMSCs highly expressed for CD73, CD90, CD29, and CD105 but negative for CD79a, CD34, CD45, CD14, and HLA-DR.

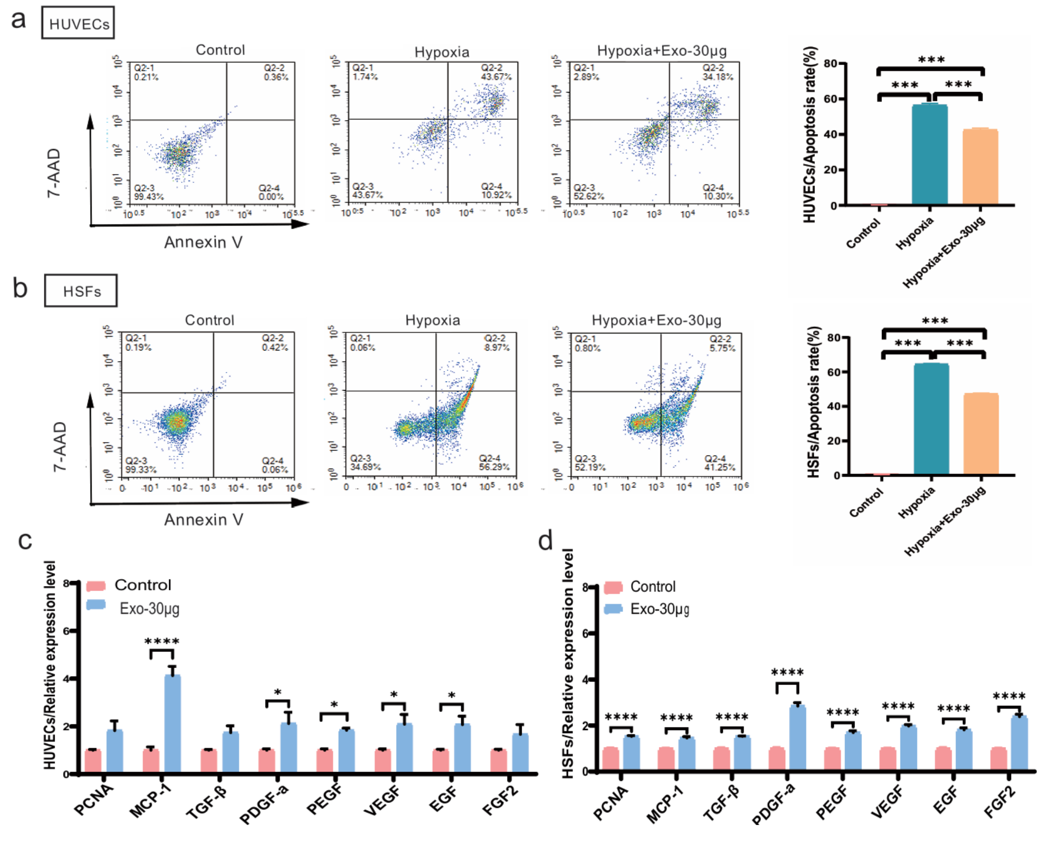

Figure S2. Anti-apoptosis and regenerative repair effects of hUCMSC-Exos. (a, b) Anti-apoptotic effect of hUCMSC-Exos on HUVECs and HSFs. Control group (treated with normoxic and without Exos), Hypoxia group (treated with hypoxia and without Exos), Hypoxia+Exo-30μg group (treated with hypoxia and with 30μg Exos). (c, d) hUCMSC-Exos affect the expression of regenerative genes in HUVECs and HSFs. Control group (without Exos), Exos group (with 30μg Exos). Results are presented as Mean ± SEM; n=3 for each group. *p<0.05, **p<0.01, ***p<0.001, ****p< 0.0001.

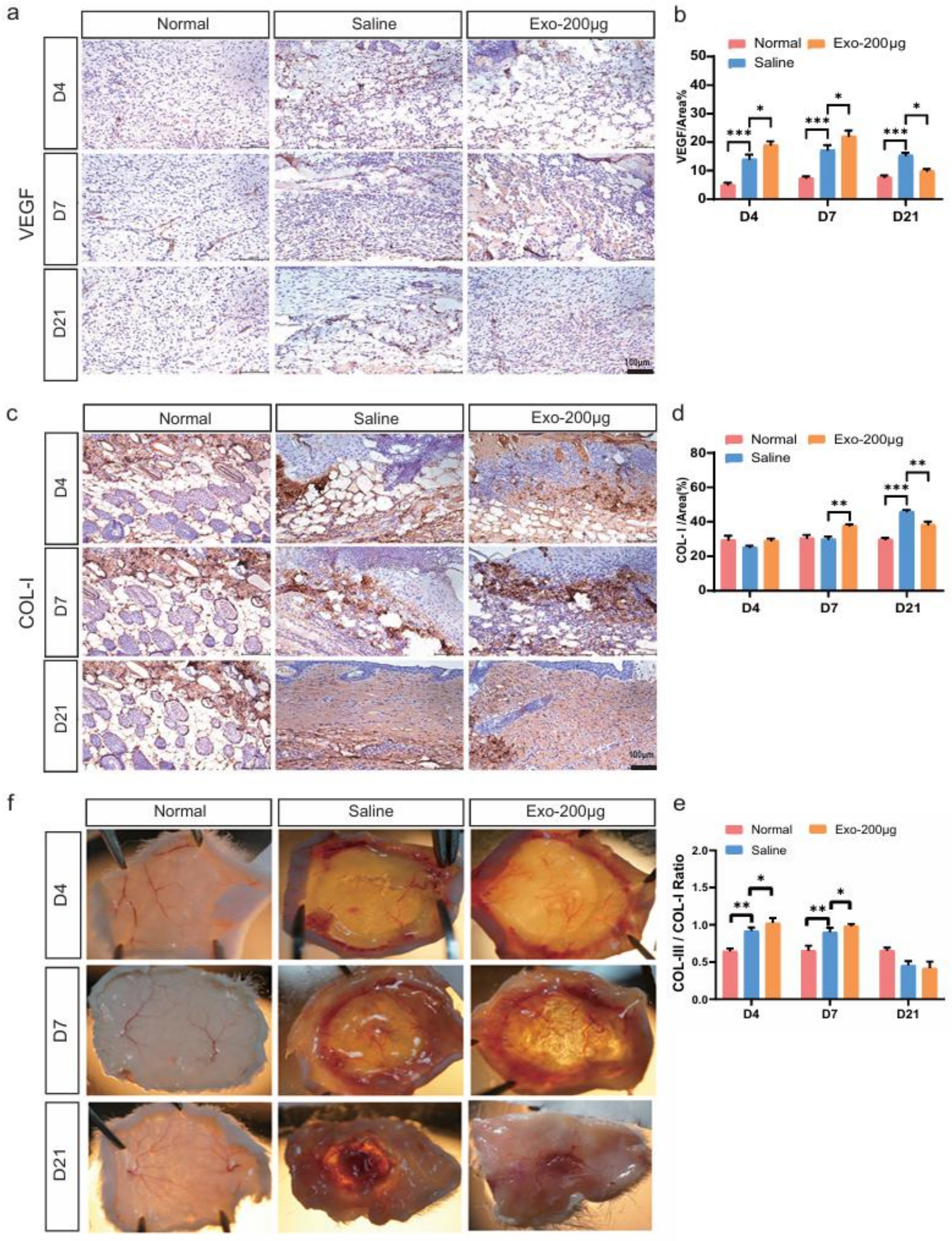

Figure S3. hUCMSC-Exos can promote angiogenesis and collagen production in damaged skin. (a-e) Immunohistochemistry staining and quantitative analysis of VEGF and collagen I expressions, and COL-III/ COL-I Ratio in skin wound tissues in different groups of mice on days 4, 7, and 21. Scale bar =100μm. (f) Dorsal skin angiogenesis in different groups of mice on days 4, 7, and 21. Results are presented as Mean ± SEM; n=6 for each group. Scar bar =100μm. *$p< 0.05$, **$p< 0.01$, ***$p< 0.001$.

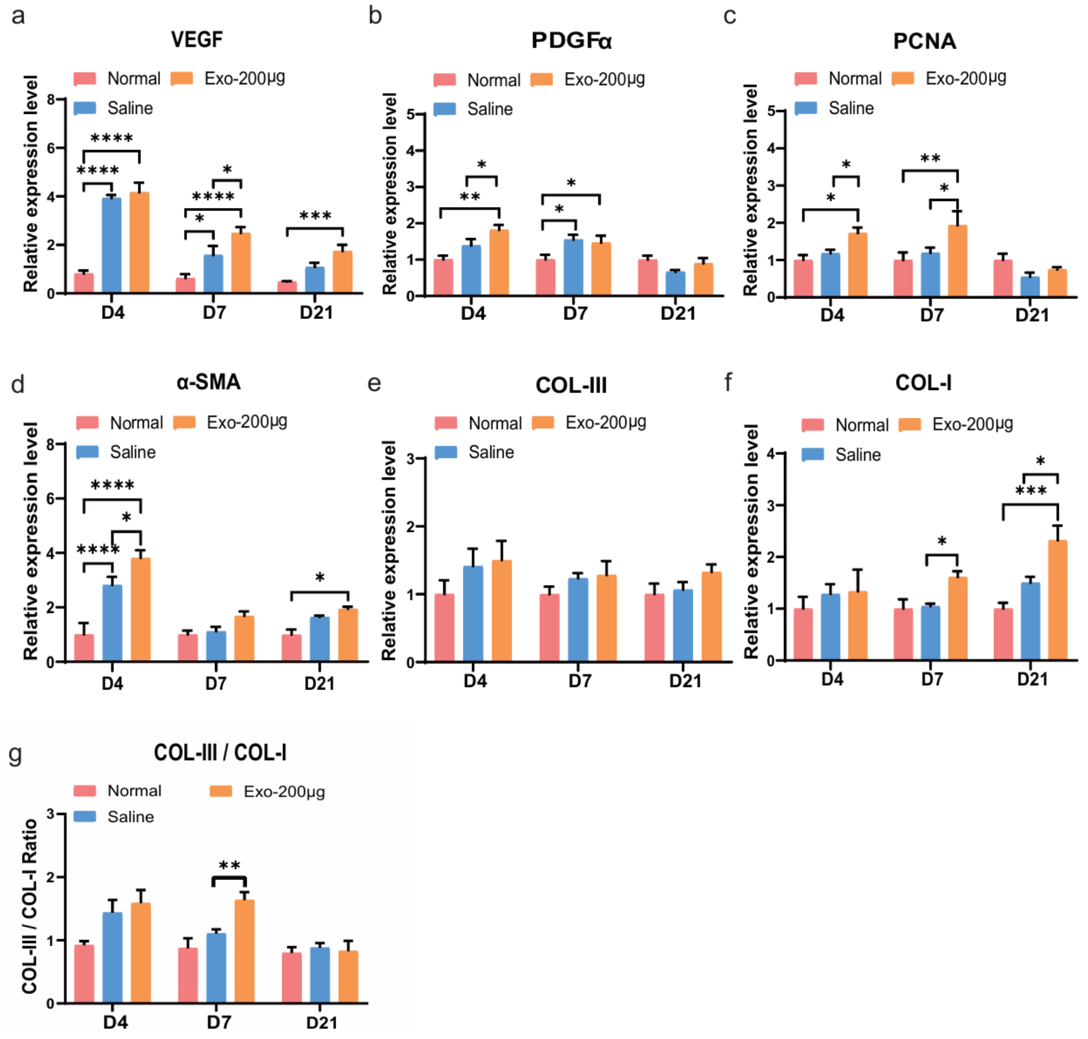

Figure S4. hUCMSC-Exos promote the expression of related regenerative genes in damaged skin. (a-g) qRT-PCR analysis of a gene expression cluster in wound tissue in each group. GAPDH served as an internal control. Results are presented as Mean ± SEM; n=6 for each group. *p < 0.01, **p < 0.01; ***p < 0.001, ****p < 0.0001.

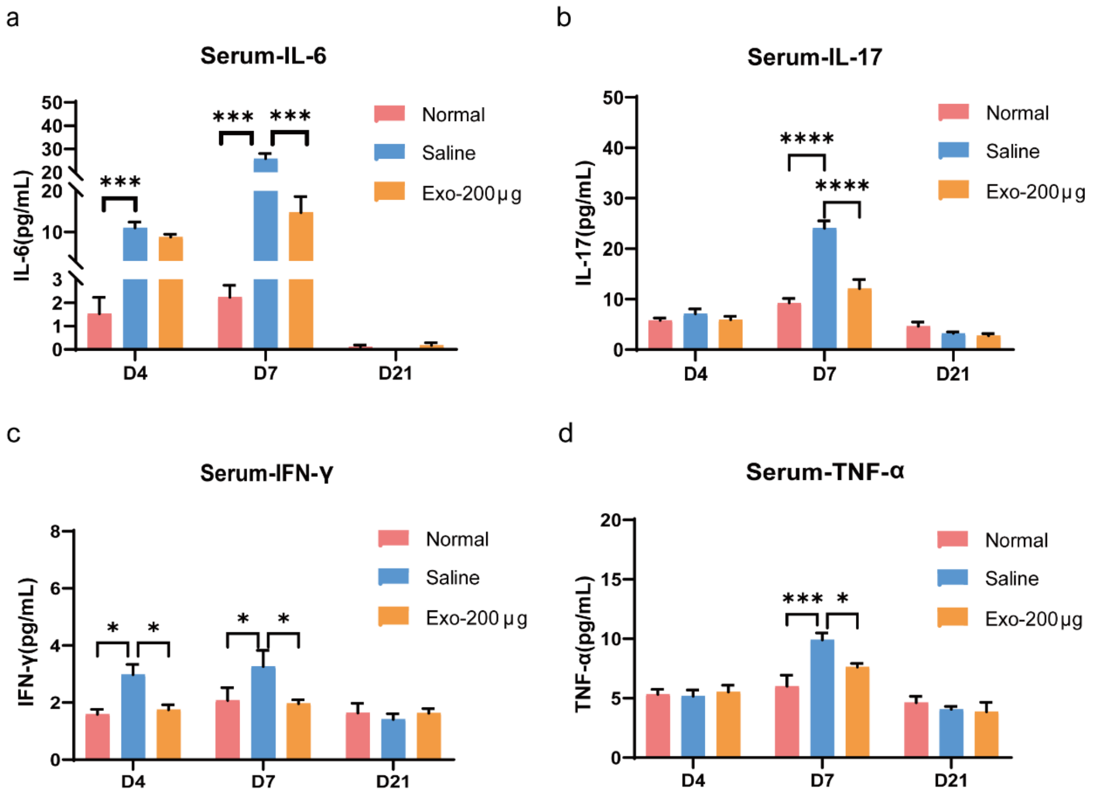

Figure S5. hUCMSC-Exos affects the expression of inflammatory factors in serum of mice. (a-d) The level of IL-6, IL-17, IFN-γ, TNF-α in serum of mice was detected by ELISA. Results are presented as Mean ± SEM; n=6 for each group. *p < 0.05; ***p < 0.001, ****p < 0.0001.

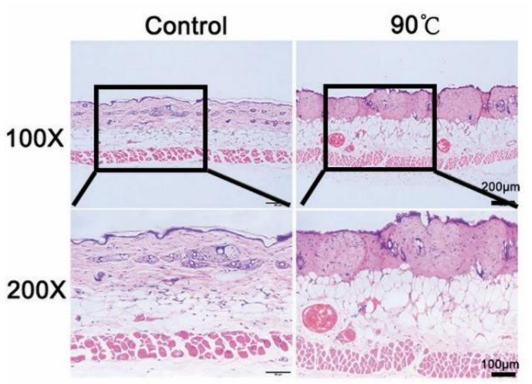

Figure S6. Histological assessment by H&E staining within 24 hours after scalding of the back skin of mice. (a) Representative images of wounded skin sections from different groups were evaluated by H&E staining within 24 h, scale bar = 200 μm in the 100 × images (top panel); scale bar = 100μm in the 200 × images (bottom panel).
